# Issues in the construction of wealth indices for the measurement of socio-economic position in low-income countries

**DOI:** 10.1186/1742-7622-5-3

**Published:** 2008-01-30

**Authors:** Laura D Howe, James R Hargreaves, Sharon RA Huttly

**Affiliations:** 1 Department of Epidemiology and Population Health, London School of Hygiene & Tropical Medicine, London, UK

## Abstract

**Background:**

Epidemiological studies often require measures of socio-economic position (SEP). The application of principal components analysis (PCA) to data on asset-ownership is one popular approach to household SEP measurement. Proponents suggest that the approach provides a rational method for weighting asset data in a single indicator, captures the most important aspect of SEP for health studies, and is based on data that are readily available and/or simple to collect. However, the use of PCA on asset data may not be the best approach to SEP measurement. There remains concern that this approach can obscure the meaning of the final index and is statistically inappropriate for use with discrete data. In addition, the choice of assets to include and the level of agreement between wealth indices and more conventional measures of SEP such as consumption expenditure remain unclear. We discuss these issues, illustrating our examples with data from the Malawi Integrated Household Survey 2004–5.

**Methods:**

Wealth indices were constructed using the assets on which data are collected within Demographic and Health Surveys. Indices were constructed using five weighting methods: PCA, PCA using dichotomised versions of categorical variables, equal weights, weights equal to the inverse of the proportion of households owning the item, and Multiple Correspondence Analysis. Agreement between indices was assessed. Indices were compared with per capita consumption expenditure, and the difference in agreement assessed when different methods were used to adjust consumption expenditure for household size and composition.

**Results:**

All indices demonstrated similarly modest agreement with consumption expenditure. The indices constructed using dichotomised data showed strong agreement with each other, as did the indices constructed using categorical data. Agreement was lower between indices using data coded in different ways. The level of agreement between wealth indices and consumption expenditure did not differ when different consumption equivalence scales were applied.

**Conclusion:**

This study questions the appropriateness of wealth indices as proxies for consumption expenditure. The choice of data included had a greater influence on the wealth index than the method used to weight the data. Despite the limitations of PCA, alternative methods also all had disadvantages.

## Introduction

### Defining and measuring socio-economic position

Socio-economic position (SEP) is a concept widely used in epidemiological research. Definitions vary, but commonly incorporate physical resources, social resources, and status within a social hierarchy[1]. Measurement of SEP is crucial not only for studies focusing on the social determinants of health, but also for the vast majority of observational health research, since SEP is likely to confound many relationships.

Traditionally, indicators of SEP have tended to be monetary measures such as income or consumption expenditure, based on the assumption that material living standards largely determine well-being[2]. Whilst it is now widely recognised that monetary measures of SEP fail to capture all of the diverse aspects of well-being, their use remains widespread, partially due to difficulties in measuring more complex conceptualisations of SEP, and because monetary measures may have clearer policy implications. There is longstanding debate about whether income or consumption expenditure is a better measure of SEP. Income is generally more variable than consumption; Friedman's permanent income hypothesis states that households are likely to base their consumption decisions on more than just their current income – people tend to 'smooth' their consumption in times of income fluctuation, for example by borrowing or drawing on savings in times of low income[3]. It is therefore widely asserted that consumption expenditure is a better marker of long-term SEP than income. This argument holds particularly strongly in low-income countries, where income may come from a variety of sources and may vary dramatically across seasons. Longer-term aspects of SEP are thought to be most relevant to many health outcomes, adding to the reasons for choosing consumption expenditure over income.

In low-income countries, measurement of consumption expenditure is fraught with difficulties. There are problems with recall and reluctance to divulge information. Additionally, prices are likely to differ substantially across times and areas, necessitating complex adjustment of expenditure figures to reflect these price differences[4]. Furthermore, collecting consumption expenditure data requires lengthy questionnaires that must be completed by skilled and trained interviewers. There are therefore both reliability and cost/time reasons why epidemiologists conducting health research in low-income countries may wish to use an alternative measure of SEP. Additionally there are existing datasets rich in health data, such as the Demographic and Health Surveys (DHS), which lack information on income or consumption expenditure.

### The asset-based approach to measuring socio-economic position

An asset-based approach to measuring household SEP is one alternative to income and consumption expenditure. This approach has arisen from demographic studies such as the DHS, which although lacking data on income or consumption expenditure, collect information on ownership of a range of durable assets (e.g. car, refrigerator, television), housing characteristics (e.g. material of dwelling floor and roof, toilet facilities), and access to basic services (e.g. electricity supply, source of drinking water). These items were all originally included in the surveys for their direct influences on health; for instance, television and radio ownership was of interest to identify households receiving public health messages. Researchers began to see that these assets could be used as indicators of living standards and have sought to construct wealth indices for that purpose[2,5]. Wealth indices measure SEP at the household level and can only be used to assess relative SEP within a population.

Collection of asset data has been claimed to be more reliable than income or consumption expenditure, since it uses simple questions or direct observation by the interviewer and should therefore suffer from less recall or social desirability bias[6]. This claim has, however, been questioned by a recent study which demonstrated at best moderate inter-observer and between-test reliability for asset data collection[7].

An asset-based wealth index could be theorised to represent long-term SEP in a similar way to consumption expenditure; asset ownership is likely to be based at least partially on economic wealth and household assets are unlikely to change in response to short-term economic shocks. There is, however, continuing debate about the appropriateness of considering a wealth index as a proxy for consumption expenditure. Two separate studies have demonstrated weak correlation between consumption expenditure and wealth indices: a study in Mozambique showed a Spearman's rank correlation coefficient of 0.37[8], and a study using multiple datasets producing R^2 ^values from regressions of consumption expenditure on a wealth index of ≤ 0.23[9]. A study using Indonesian data found that there was considerable re-ranking of households between a wealth index and consumption expenditure, with approximately 50% of households being misclassified when the population was split into the bottom 30%, middle 40% and top 30%[10]. Other studies have demonstrated considerable variation in the correlation across countries, with Spearman's rank correlation coefficients between 0.43–0.64 in one study and 0.39–0.71 in another[6,11]. It could be argued that a wealth index captures a longer-term state of wealth than consumption expenditure; in times of economic shock, selling assets is likely to come subsequent to reductions in consumption expenditure. As both measures attempt to measure long-term SEP, and since it is useful to have a standard against which to judge wealth indices, we will consider consumption expenditure as a gold standard measure of long-term SEP, and explore the extent to which wealth indices agree with consumption expenditure.

### Weighting the items in a wealth index

When constructing a wealth index from a set of variables, a decision must be made about the weights to assign to each indicator. Principal Components Analysis (PCA) was recommended as a method for determining weights for components of a wealth index by Filmer and Pritchett[11]. Guidelines for the use of PCA for wealth indices were published by Vyas and Kumaranayake[12].

PCA is a 'data reduction' procedure. It involves replacing a set of correlated variables with a set of uncorrelated 'principal components' which represent unobserved characteristics of the population. The principal components are linear combinations of the original variables; the weights are derived from the correlation matrix of the data or the covariance matrix if the data have been standardised prior to PCA. The first principal component explains the largest proportion of the total variance. If the first few principal components explain a substantial proportion of the total variance, they can be used to represent the original items, thus reducing the number of variables required in models[13].

For constructing a wealth index, the first principal component is taken to represent the household's wealth[14]. The weights for each indicator from this first principal component are used to generate a household score. Assets that are more unequally distributed across the sample will have a higher weight in the first principal component[12]. The relative rank of households using the score generated from the first principal component is then used as a measure of relative SEP, enabling calculation of a single estimate of the effect of wealth[15]. The use of a single principal component in this way could be questioned, since the first principal component from PCA of a set of assets frequently explains a low proportion of the total variation in those assets (often less than 20%)[11,12,16]. It could be the case that the theoretical 'wealth' construct is multi-dimensional, with the first few principal components each capturing a specific aspect of wealth. Using only the first principal component would, in this case, not capture the entire wealth effect. However, the aim of using PCA to generate a wealth index is to define a single indicator of SEP, and using multiple principal components would not be compatible with this. If the first principal component explains a small proportion of the total variance, each subsequent higher order component will explain a smaller proportion still, so using two or three principal components may not drastically improve the proportion of the total variance explained. It is also not generally straightforward to identify which aspects of wealth higher order principal components might represent, since there is not usually a clear pattern of which assets are assigned positive/negative or higher/lower weights. Furthermore, there is some evidence that utilising higher order principal components is unnecessary. McKenzie demonstrated that the standard deviation of higher order components was not associated with consumption expenditure, whereas that of the first principal component was[16]. Filmer and Pritchett noted that multivariate analyses of the association between the wealth index and school enrollment were robust to the inclusion of higher order components[11].

After the paper by Filmer and Pritchett, the use of PCA for wealth index construction was quickly adopted by the World Bank and Macro International Inc. for analysis of inequalities within DHS datasets[5,17-19]. The approach is now also more widely used. Nevertheless, this application of PCA is not fully justified and requires further investigation. PCA is designed for use with continuous, normally-distributed data. Its application to the predominantly discrete data in a wealth index is therefore inappropriate. The use of binary dummy variables for each category of categorical variables (as recommended by Filmer and Pritchett[11]) is particularly problematic. The linear dependence between the dummy variables may lead to incorrect estimates of the wealth index; the PCA method is affected by collinearity, with variation in the data arising both from the underlying concept of wealth and from the linear dependence between dummy variables of categorical variables. This approach has been shown to be inferior to several alternative methods of dealing with categorical data[20]. The alternative methods explored were using ordinal variables, using group means, and using polychoric correlations. These methods, whilst being preferable in terms of the data assumptions of PCA, do require strong assumptions about the ordinal nature of the data. It is not necessarily straightforward, for instance, to rank different sources of drinking water, and to assume that they are equally spaced from each other in terms of their relationship with SEP.

The limitations of PCA for the construction of wealth indices are thus twofold: i) PCA is problematic with the discrete data commonly included in a wealth index, and ii) the first principal component frequently explains only a low proportion of the total variation in asset data. Furthermore, PCA is a fairly complex method. It is likely to be unfamiliar and poorly understood by less technical readers of papers. It could therefore be argued that simpler, more familiar and easily understood methods for weighting the items in a wealth index would be preferable. Using an equal weights approach (simple sum) was used in several early studies using wealth indices[21,22]. Although simple, this approach could be criticised for being arbitrary and simplistic, since different assets are unlikely to have equal meaning in terms of SEP. The literature comparing indices constructed using PCA and using an equal weights approach is not consistent. There is some evidence that PCA performs no better as a proxy for consumption expenditure than an equal weights approach[23]. In contrast, Bollen *et al*. showed that a PCA-based wealth index and an equal weights index had considerably different regression coefficients with consumption expenditure[24]; another study also demonstrated that a PCA-based wealth index had a stronger relationship than an equal weights index with a latent variable of permanent income (planned and anticipated income, a long-term concept of SEP that both consumption expenditure and wealth indices have been claimed to be measuring)[25].

Another potentially simpler and more easily understood alternative to PCA is to use the inverse of the proportion of households that own an asset as its weight. This is based on a method originally suggested by Townsend[26]. The underlying assumption is that assets owned by a smaller proportion of households are indicative of higher household wealth and are therefore assigned a higher weight[27]. A problem with methods using inverse proportion weights is that not all assets show a linear relationship with living standards, e.g. ownership of a motorbike may tend to increase up to a certain income and subsequently decrease in richer households[5]. A similar method was applied by Morris *et al*., who calculated weights by using the inverse of the proportion of households that owned each item, multiplying that by the number of units of asset owned by the household, and summing this quantity for all assets[28]. Both the equal weights and the inverse proportion weighting methods can only be applied to binary data.

Multiple Correspondence Analysis (MCA) is analogous to PCA, but is for discrete data[29]. Whilst this method does not remove the complexity and unfamiliarity of PCA, nor the problems of the first dimension explaining a small proportion of the total variance, it is appropriate for the analysis of the categorical data commonly collected on most assets[30]. Booysen *et al*. utilised MCA to construct wealth indices for seven sub-Saharan African countries. They found that the index was very highly correlated with one constructed using PCA, and that although households were not always in the same quintile by the two indices, movement was in most cases limited to one quintile in either direction. They also showed that the weights assigned to index items were generally similar by the two methods[30].

Other methods for weighting items in a wealth index do exist, but in general offer neither more simplicity than PCA, nor more suitability for discrete data. For instance, latent variable approaches have been proposed[31,32]. In his 2005 paper, Montgomery constructs a wealth index using a latent variable approach called MIMIC; this model specifies which variables are determinants of living standards (e.g. education and occupation) and which are indicators of living standards (e.g. consumer durables). In other methods of wealth index construction, both determinants and indicators of the underlying socio-economic construct may be included without distinction. For instance, producer durables such as farm equipment are sometimes included in a wealth index in the same way as consumer durables, whereas these should in fact be considered as determinants of the socio-economic construct and not treated in the same way as indicator variables[31]. Latent variable methods, despite offering some theoretical advantages over PCA, are far more complex and arguably even less easily understood by a wide readership than PCA. A further option could be to assign weights based on the price of an item, but this requires detailed information allowing for date of purchase, area of purchase, and current condition of the item. There is also some evidence that price-based indices are less reliable than alternatives; one study showed a price-based index to have implausible relationships with health outcomes[33] and a further study demonstrated that two price methods had weaker relationships with a permanent income latent variable than alternative weighting methods[25]. In contrast, however, Morris *et al*. showed high correlation between wealth indices constructed using the inverse proportion method and weights based on the current value of each item[28]. The issue of prices is a crucial one. Consumption expenditure measures are adjusted for the variability of prices across regions. In contrast, the variability in prices is generally ignored when pooling data across regions to construct a wealth index. The methods currently used in the literature to incorporate prices into weights for wealth index indicators (typically relying on self-reported current sale value) do not, however, appear to be appropriate, and more complex methods involving regional price data calculation similar to the approach used for consumption expenditure data would probably be too costly for the majority of epidemiological studies.

### Which concept of long-term SEP does a wealth index represent?

Both consumption expenditure and wealth indices are measured using household-level data. Equivalence scales are generally applied to consumption expenditure data in order to allow for household size and composition. The most frequently used equivalence scales are per capita (i.e. divided by the total number of household members), per adult or per adult equivalent (where each child is considered to require a pre-determined proportion of the consumption of one adult). Wealth indices, however, are not generally adjusted for household size or composition. There is some evidence that adjusting a wealth index for household size results in implausible relationships with health outcomes[5]. It has also been argued that while consumption needs and patterns will obviously be strongly affected by household size and composition, the benefits of most items included in a wealth index are at the household level[5]. It has, however, been demonstrated that wealth indices and per capita expenditures produce very different patterns in household size; in 11 low-income countries, the poor-rich difference in average household size was consistently greater when using per capita expenditures compared with a wealth index[34]. This indicates that households with a greater number of members, a factor often associated with poverty, would not always end up in the lower quintiles of a wealth index.

In considering the appropriateness of a wealth index as a proxy for consumption expenditure, it has been suggested that the choice of equivalence scale may have a substantial impact on the observed relationship. Sahn and Stifel suggested that the correlation of a wealth index would be highest when total household expenditures were considered, intermediate when a per adult equivalence scale is used, and lowest when per capita consumption expenditure is used[6]. There is, however, no evidence of this presented in the current body of literature.

### Aim

The aim of these analyses is to compare wealth indices constructed using different weighting methods to identify whether PCA offers an advantage over either simpler, more transparent methods (equal weights and inverse of the proportion of the population owning the asset) or methods more appropriate for discrete data (MCA). Furthermore, the agreement of a wealth index with consumption expenditure measures adjusted for household size and composition in different ways will be examined to identify which aspect of long-term SEP a wealth index best represents.

## Methods

To illustrate our exploration of wealth indices, we analysed data from the Malawi Integrated Household Survey 2004–5 (IHS2)[35]. This national survey of 11,280 households collected data on the socio-economic living conditions in Malawi. It contained both asset data and a measure of consumption expenditure. The measure of consumption expenditure was calculated using annualised figures for consumption expenditure across categories of food- and non-food consumption according to the UN classification system 'Classification of Individual Consumption According to Purpose'. A price index was used to adjust for differences in prices across areas and times. The Malawi National Statistical Office evaluated equivalence scales for the consumption expenditure aggregate, and found the poverty profile to be remarkably similar when a per capita or a per adult equivalent scale was used[36]. For these analyses, a per capita equivalence scale was used, i.e. total household consumption expenditure was divided by the number of household members. The assets used to construct the wealth indices were those used in analyses by the World Bank of the 2000 Malawi DHS (toilet facility, main cooking fuel, main drinking water source, floor material of main dwelling, whether there is electricity in the home, owns radio, owns television/VCR, owns bicycle, owns car, owns motorbike/scooter, owns agricultural land, and presence of a domestic servant)[19]. All data cleaning and analyses were performed in Stata version 9[37].

Wealth indices were constructed using the following methods to weight data:

1. Using PCA including all categories of categorical variables

2. Using PCA but with dichotomised versions of all categorical variables

3. Applying equal weights to binary variables

4. Weighting binary variables by the inverse of the proportion of the population which owns that item

5. Using MCA including all categories of categorical variables

Following recommended practice, for index 1 dummy binary variables were created for each category of categorical variable for inclusion in the PCA; for example a four-category variable would have been converted into four separate yes/no variables; for each household one of these would be coded 'yes' the other three 'no'[12]. Alternative ways of using categorical variables in PCA were not used because they require imposing an ordinal structure on the categories.

Applying equal weights and using the inverse of the proportion of the population that owns the item can only be carried out using binary variables. Therefore, for the purposes of creating indices 3 and 4, each categorical variable was collapsed to a binary variable based on a subjective assessment of the most appropriate dichotomisation, resulting in an appropriate distribution of ownership and meaningful categories. The detailed entries for observations coded as 'other' were examined in order to determine the most appropriate way to classify the 'other' group. The dichotomisations are detailed below:

### Details of dichotomisation of categorical variables

#### Floor material

• Lower SEP group: sand, smoothed mud

• Higher SEP group: smooth cement, tile, other

#### Cooking fuel

• Lower SEP group: firewood, crop residue, other

• Higher SEP group: paraffin, electricity, charcoal

#### Water supply

• Lower SEP group: personal open unprotected well, communal open unprotected well, river, spring, lake, reservoir, other

• Higher SEP group: piped into dwelling, piped outside dwelling, communal standpipe, personal handpump, communal handpump, protected spring

#### Toilet facility

• Lower SEP group: no toilet facility, other

• Higher SEP group: flush toilet, VIP latrine, traditional latrine with roof, latrine without roof

In addition to using these binary variables for indices 3 and 4, index 2 was created in order to explore its agreement with index 1, and to facilitate a more direct comparison of the PCA approach with the simpler weighting methods used in indices 3 and 4.

Indices were standardised to give a mean of zero and a variance of one. Survey analysis was used for descriptive analyses to adjust for the complex sampling used in IHS2. Sampling weights cannot be applied during MCA and PCA; therefore, in order to facilitate comparisons, sampling weights were not used when calculating the weights for any index, but they were used for generating quintiles, as in previous studies[19,38].

The PCA-based indices utilised the weights from the first principal component to ascertain the weights.

A Stata macro for MCA was downloaded from the EconPapers website[39]. In a similar manner to PCA, the weights used are those identified from the first dimension of the MCA. However, unlike PCA, the MCA command is not compatible with post-estimation commands in Stata. Thus, in order to apply the weights, a score variable was manually generated applying the appropriate weight from the MCA to each indicator.

The distribution of each index was examined graphically to assess the extent of skewness and clumping. Clumping is a problem commonly found in wealth indices whereby a large proportion of households have the same (usually low) score, because a large number of households have similar (low) access to public services and ownership of consumer durables.

Indices were compared with each other in terms of scatter diagrams and misclassification of households between quintiles of indices. Kappa statistics were calculated in order to assess the agreement of classification between indices. The Kappa statistic is a measure of reliability that takes into account the agreement expected on the basis of chance. A Kappa statistic of one indicates perfect agreement and a value of zero indicates no agreement better than chance. There are no universal rules for interpreting Kappa statistics, but in general a value of less than 0.5 would indicate poor agreement. Misclassification between quintiles was chosen as the measure of agreement since almost all epidemiological studies using a wealth index will use quintiles of the index in analyses. Although previous studies have often used correlation coefficients to compare indices, this can be misleading since correlation can hide a systematic bias and does not necessarily imply agreement. Graphs were also constructed to compare indices; scatter plots were used for comparing two indices both using categorical data, and box-plots were used when one or both of the indices used binary variables.

In addition to comparisons between the indices, each index was compared with per capita consumption expenditure, which despite having its own limitations and reliability issues was taken as a gold standard measure of SEP.

In order to assess which aspect of long-term SEP a wealth index best represents, consumption expenditure measures were constructed adjusted in the following ways: i) no adjustment, i.e. total household expenditures, ii) per adult expenditures and iii) per capita expenditures. The agreement of each consumption expenditure measure with a wealth index was calculated. The wealth index was constructed from the same asset indicators as above, using PCA.

## Results

Missing data levels were very low. Complete data were available on 11,243 of 11,280 households (99.7%).

### Distribution of Indices

**Figure 1:**
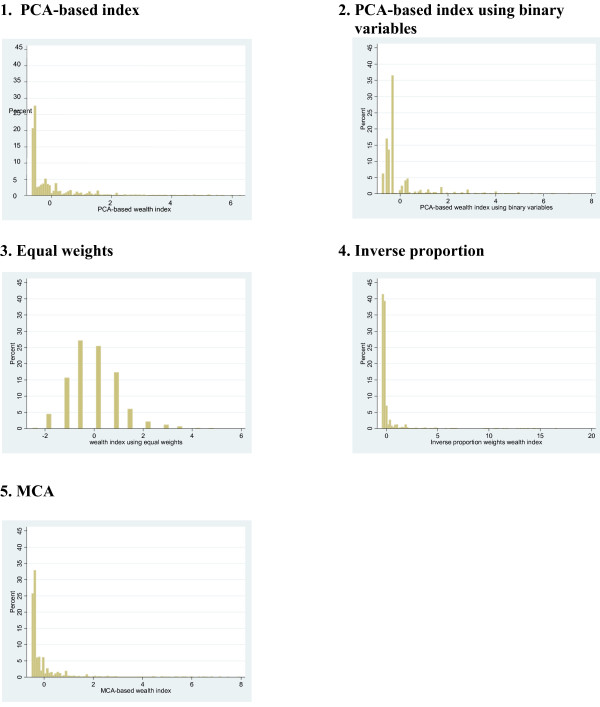


Figure 1 shows histograms of the five wealth indices. Apart from Index 3 (equal weights), all indices were highly right-skewed. Index 3 was less skewed, but had severe clumping, with the score taking just 20 unique values compared with several thousand for the other indices. All indices demonstrated clumping, with many households having the same or very similar scores at the lower end of the spectrum. Clumping was more severe in indices using binary variables, with indices 2 and 4 demonstrating more clumping than indices 1 and 5.

### Weights assigned to index components

Tables 1 and 2 show the weights assigned to each indicator in the five indices. All weights are in the expected directions (i.e. items expected to be associated with higher SEP have a positive weight and vice versa) apart from agricultural land, which has a negative weight in the PCA and MCA indices. The negative weight assigned to agricultural land is consistent with the index used in World Bank analysis of the Malawi DHS[19]. The weights are not directly comparable between indices, as they are on different scales. However, the relative magnitude of weights across indices can be compared, and this illustrates some striking differences between the indices. For instance, the ratio of the weight assigned to a motorbike to the weight assigned to a car is 0.23 in the PCA index, 0.26 in the PCA index using binary variables, 1 in the equal weights index, 3.3 in the inverse proportion index and 0.39 in the MCA index. Thus motorbike has a proportionally far higher weight than a car in the inverse proportion index, indicative of the fact that the prevalence of motorbike ownership is very low. All item weights in indices 1 and 5 (PCA and MCA) are of very similar relative proportions.

**Table 1:** Weights assigned to each indicator in indices using categorical variables:

**Item**		**Item weight**	
		***PCA***	***MCA***
**Toilet facility:**			
Flush toilet		*0.2760*	*2.081*
VIP latrine		*0.0894*	*0.515*
Traditional latrine with roof		*0.0015*	*-0.019*
Latrine no roof		*-0.0613*	*-0.125*
None or other		*-0.0923*	*-0.197*
			
**Water source:**			
			
Piped inside dwelling		*0.2762*	*2.428*
Piped outside dwelling		*0.1631*	*0.857*
Communal standpipe		*0.1251*	*0.161*
Personal handpump or well		*0.0154*	*0.011*
Communal handpump or well		*-0.2270*	*-0.138*
River, lake, spring, reservoir, or other		*-0.0433*	*-0.179*
			
**Cooking fuel:**			
Collected firewood		*-0.3049*	*-0.153*
Purchased firewood		*0.1252*	*0.176*
Paraffin, gas or charcoal		*0.2196*	*0.721*
Electricity		*0.2451*	*2.537*
Crop residue, saw dust, or other		*0.0043*	*-0.084*
			
**Floor material:**			
Sand		*-0.0078*	*-0.168*
Smoothed mud or other		*-0.3113*	*-0.154*
Smooth cement, wood, or tiles		*0.3310*	*0.613*
**Electricity:**	Yes	*0.3427*	*1.6*
	No	*-*	*-0.1*
**Radio:**	Yes	*0.0193*	*0.007*
	No	*-*	*-0.009*
**TV:**	Yes	*0.2836*	*1.726*
	No	*-*	*-0.070*
**Bike:**	Yes	*0.0025*	*0.002*
	No	*-*	*-0.001*
**Car:**	Yes	*0.1885*	*2.247*
	No	*-*	*-0.028*
**Motorbike:**	Yes	*0.0432*	*0.869*
	No	*-*	*-0.003*
**Domestic servant:**	Yes	*0.1426*	*1.32*
	No	*-*	*-0.025*
**Agricultural land:**	Yes	*-0.2280*	*-0.081*
	No	*-*	*0.589*

**Table 2:** Weights assigned to each indicator in indices using binary variables:

**Item**	**Item weight**		
	***PCA***	***Equal weights***	***Inverse proportion***
**Toilet facility:**			
some toilet facility	*0.1429*	*1*	*1.2*
			
**Water source:**			
protected source	*0.1703*	*1*	*1.5*
			
**Cooking fuel:**			
more likely to have been purchased	*0.4320*	*1*	*11.8*
			
**Floor material:**			
modern	*0.4084*	*1*	*5.0*
			
**Electricity:**	*0.4600*	*1*	*17.1*
			
**Radio:**	*0.0225*	*1*	*1.8*
			
**TV:**	*0.4012*	*1*	*25.7*
			
**Bike:**	*0.0014*	*1*	*2.8*
			
**Car:**	*0.2766*	*1*	*82.3*
			
**Motorbike:**	*0.0725*	*1*	*275.1*
			
**Domestic servant:**	*0.2190*	*1*	*53.4*
			
**Agricultural land:**	*-0.3072*	*1*	*1.1*

### Agreement of the indices with consumption

All of the indices have similar levels of misclassification between quintiles of the wealth index and quintiles of per capita consumption expenditure, with only approximately 30% of households in the same quintile and Kappa statistics of roughly 0.1 (Table 3). Index 5 (MCA-based index) has the best agreement with per capita consumption expenditure, and Index 3 (equal weights) the worst agreement, but the differences between indices are small, indicating that their ability to proxy consumption expenditure is similarly modest.

**Table 3:** Movement of households between quintiles of wealth indices and per capita consumption expenditure

**% Households moving between quintiles of the wealth index and quintiles of per capita consumption expenditure**	**1. PCA index**	**2. PCA index using binary variables**	**3. Equal weights index**	**4. Inverse proportion index**	**5. MCA index**
Same quintile	28.9	28.0	26.6	28.2	29.2
Move one quintile	34.8	36.0	37.8	33.6	34.3
Move two quintiles	21.5	20.6	22.3	22.5	22.1
Move three quintiles	11.6	12.2	10.5	11.3	11.4
Move four quintiles	2.9	3.1	2.8	4.4	3.0
***Kappa***	0.11*	0.10*	0.082*	0.10*	0.12*

*p < 0.001

### Comparing the indices

Table 4 shows a matrix of the Kappa statistics between indices, and Table 5 tabulates the movement of households between quintiles of pairs of wealth indices.

**Table 4:** Percentage of households in the same quintile and Kappa statistics of agreement between pairs of indices

	**1. PCA**	**2. PCA (binary)**	**3. Equal weights**	**4. Inverse proportion**	**5. MCA**
**1. PCA**	-				
**2. PCA (binary)**	41.9% κ = 0.27*	-			
**3. Equal weights**	35.9% κ = 0.20*	73.6% κ = 0.67*	-		
**4. Inverse proportion**	39.3% κ = 0.24*	69.5% κ = 0.62*	67.7% κ = 0.60*	-	
**5. MCA**	75.6% κ = 0.69*	51.5% κ = 039*	40.6% κ = 0.26*	43.4% κ = 0.29*	-

*p < 0.001

**Table 5:** Movement of households between quintiles of the indices

**Wealth indices being compared**	**% Households moving between quintiles**				
	*Same quintile*	*Move 1 quintile*	*Move 2 quintiles*	*Move 3 quintiles*	*Move 4 quintiles*
Index 1 (PCA all categories) and Index 2 (PCA binary variables)	41.9	41.3	13.3	4.5	0.4
Index 1 (PCA all categories) and Index 3 (Equal weights)	35.9	38.5	18.8	7.1	1.1
Index 1 (PCA all categories) and Index 4 (Inverse proportion)	39.3	39.2	13.3	8.6	0.98
Index 1 (PCA all categories) and Index 5 (MCA)	75.6	18.9	5.8	0.65	0.33
Index 2 (PCA binary variables) and Index 3 (Equal weights)	73.6	18.7	4.5	4.0	0.5
Index 2 (PCA binary variables) and Index 4 (Inverse Proportion)	69.5	23.1	5.6	2.7	0.33
Index 2 (PCA binary variables) and Index 5 (MCA)	51.5	36.3	11.6	1.5	0.36
Index 3 (Equal weights) and Index 4 (Inverse proportion)	67.7	28.8	3.5	0.91	0.37
Index 3 (Equal weights) and Index 5 (MCA)	40.6	38.4	16.4	4.9	1.0
Index 4 (Inverse proportion) and Index 5 (MCA)	43.4	39.8	10.5	6.7	0.90

Comparing Index 1 (PCA) and Index 5 (MCA), which both used categorical variables, approximately 75% of households were in the same quintile in the two indices, with a Kappa statistic of 0.69. For households in different quintiles, movement was generally limited to one quintile, with less than 5% of households moving two or more quintiles.

Agreement between pairs of indices using binary variables (Indices 2, 3 and 4) was also reasonably high, with approximately 70% of households being in the same quintile between two indices and Kappa statistics of approximately 0.6.

When comparisons were made between an index using categorical variables and an index using binary variables, agreement was weaker. Here, approximately 35–50% of households were in the same quintile between pairs of indices, with Kappa statistics of 0.2–0.4.

**Figure 2:**
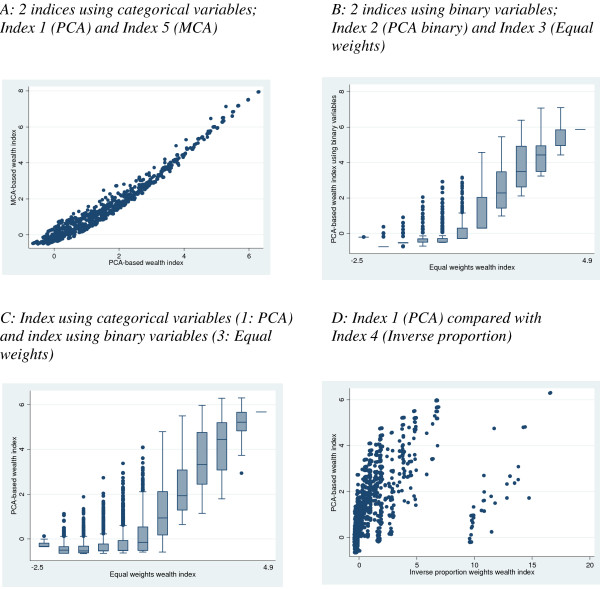


Figure 2 shows diagrams of the relationship between selected pairs of indices to illustrate key points. These diagrams demonstrate that indices constructed by different weighting methods but using the same form of data (i.e. comparing two indices using categorical variables or comparing two indices using binary variables – Figures 2A and 2B) showed a reasonably good relationship in comparison with the relationship between pairs of indices constructed using different data (i.e. comparing an index using categorical variables with an index using binary variables – Figure 2C), which showed considerably more scatter. The scatter between the indices using categorical variables (Figure 2A) was markedly less than the scatter between the indices using binary variables (Figure 2B).

Figure 2D demonstrates that Index 4 (Inverse proportion) created a group of outliers; households which were ranked substantially higher by the inverse proportion index than by the PCA index. This pattern was present in comparisons of the inverse proportion index with all other indices. Closer examination of this group of households reveals that they have a significantly higher prevalence of motorbike ownership; 52.6% of households with a score of > 9 on the inverse proportion index own a motorbike, compared with 0.36% in the whole population. This demonstrates that when items of very low prevalence are included in an index constructed using the inverse proportion weighting method, the resultant very high weight they are assigned can produce some strange classifications of households.

The agreement of the wealth index did not differ to any substantial degree when different equivalence scales were used for consumption expenditure (Table 6). The percentage of households in the same quintile was highest for per capita consumption expenditure (28.9%) but the difference between this and the agreement with total (28.8%) and per adult (27.3%) consumption expenditure was small.

**Table 6:** Agreement of the wealth index with different measures of consumption expenditure

	**% Households moving between quintiles of wealth index and per capita consumption expenditure**					
**Consumption equivalence scale**	*Same quintile*	*Move 1 quintiles*	*Move 2 quintiles*	*Move 3 quintiles*	*Move 4 quintiles*	**Kappa (SE)**

Total consumption expenditure	28.8	34.7	21.7	12.1	2.7	0.10 (0.005)
Per adult consumption expenditure	27.3	35.7	21.1	12.8	3.0	0.090 (0.005)
Per capita consumption expenditure	28.9	34.8	21.5	11.6	2.9	0.11 (0.005)

## Discussion

The use of PCA to assign weights to assets included in a wealth index has gained popularity in recent years. Despite this popularity, this application of PCA remains novel; it is statistically unsuitable for use with the categorical data frequently included in wealth indices, and has not been fully investigated. Simpler, more familiar and easily understood methods for weighting a wealth index could include assigning equal weights to all items, or using weights equal to the inverse of the proportion of households owning the item.

We have shown that within this context, the way data are coded is far more important than the weighting method used to construct the index. Indices using data coded in the same way demonstrated high agreement with each other. Agreement was considerably lower between wealth indices constructed using data coded in different ways, i.e. indices using categorical variables compared with indices using binary variables. This suggests that the indicators used in a wealth index are of great importance, although further work attempting to replicate this finding in other settings would be beneficial. Whilst these analyses have used only the assets collected by DHS, further work investigating the effects of using a wider/different set of assets is recommended. Bollen *et al*. showed that within the Ghana 1998/9 Living Standards Measurement Study (LSMS), a wealth index constructed using a wider set of indicators had a stronger relationship with a permanent income latent variable than a wealth index constructed using only the core set of assets included in the DHS; in the Peru 1985 LSMS, however, the difference was small[25]. Researchers are urged to remember that this set of core assets was not originally included in the DHS for SEP measurement; the assets predictive of wealth may vary substantially between settings and over time and if the wealth index approach to SEP measurement is used in new data collection, it would seem unwise to rely on this set of assets without further exploration of the important indicators of SEP in a particular context.

The fact that the core set of assets in the DHS were originally included in the surveys for their direct effects on health has additional implications. Depending on the outcome of interest, many indicators commonly included in a wealth index potentially have direct effects on health. It may be the case, therefore, that variables are 'double counted' if included both in a wealth index and as separate indicators in a model, making interpretation of coefficients unclear. Houweling *et al*. demonstrated that excluding from the wealth index variables thought to have the strongest direct effects on child health did affect the magnitude and even direction of inequalities in child health, but the effect was not consistent across countries[38]. One approach to disentangle the effects of education on child health has been to include the education of the household head in the wealth index, and use the education of the child's parents as separate variables[31].

In analyses such as ours, which use large existing datasets, application of an inverse proportion approach can lead to items that are meaningless in a given context being assigned a large weight. This is demonstrated in our analyses by the fact that ownership of a motorbike was assigned a very high weight in the inverse proportion index, far higher than car ownership. In the other indices, car ownership is assigned a higher weight than motorbike ownership, as would probably be expected. This resulted in a sub-set of households being ranked far higher by the inverse proportion index than by the other indices. We would therefore suggest that using the inverse proportion weighting method is only suitable when data collection has been informed by formative research.

The indices all had similarly modest agreement with consumption expenditure. Within this setting, neither the weighting method used to construct the index nor the difference between using categorical and binary variables has a strong impact on the ability of a wealth index to proxy consumption expenditure. The modest agreement with consumption expenditure brings into question the use of a wealth index as a proxy for consumption, and raises the question of what a wealth index should be considered to be measuring. Despite its use in this and other studies as a gold-standard measure of SEP, consumption expenditure itself has considerable limitations and reliability issues. The lengthy questionnaires requiring accurate details of expenditures on many items over varying periods mean that the variable is at risk from substantial measurement error. Furthermore, the adjustments required for price differences across regions and imputations for rental value of housing and use-value of durable goods require considerable assumptions and therefore introduce the possibility of bias. Consumption expenditure itself could be viewed as a proxy for some underlying socio-economic concept, such as Friedman's notion of permanent income – planned and anticipated income, as opposed to current income[3]. The wealth index may therefore be measuring a different aspect of this underlying socio-economic concept than consumption expenditure, or it may be measuring something else entirely. Some have claimed that a wealth index measures a longer-term economic status than consumption expenditure, since households are more likely to alter consumption in response to an economic shock than they are to sell assets or alter housing characteristics or access to public services[11]. In this context, the agreement of the wealth index with consumption expenditure did not differ between total, per adult and per capita consumption expenditure, meaning that this study was unable to shed further light on which aspect of long-term SEP a wealth index may be measuring.

The appropriateness of the wealth index as a measure of SEP may differ between sub-groups of the population; different household economic strategies may affect the proportion of income that is spent on consumer durables. For instance, city slum-dwellers may be at risk of frequent relocation and theft, and may therefore choose not to invest in durable goods, perhaps resulting in a lower wealth index score than may be appropriate. In addition, because prices are not generally taken into consideration in wealth index construction, the appropriateness of the wealth index may differ between urban and rural areas, and between regions. Further research into the extent of these differences and strategies to overcome them is warranted.

In terms of the ability of a wealth index to proxy consumption expenditure, PCA appears to offer little advantage over the simpler, more easily understood methods, nor over the more statistically appropriate method of MCA. However, agreement between the indices using the categorical variables and the indices using the binary variables was modest, suggesting that the data included in the wealth index does impact on the final index. While it is not possible to judge whether the indices using categorical data or the indices using binary data are more appropriate based on the agreement with consumption expenditure, other features of the data can be used to make this assessment. There will inevitably be some loss of information between categorical and binary variables, and few would disagree that more detailed information is generally preferable. Decisions regarding the dichotomisation of variables will inevitably be subjective to a large degree, and may therefore be inappropriate or sub-optimal. Furthermore, the indices using categorical variables demonstrated considerably less clumping than the indices using binary variables, making it easier to generate quintiles of even size and improving differentiation between households. It could therefore be argued that PCA and MCA may be preferable over equal weights or inverse proportion approaches, despite the simple interpretation and ease of understanding for a wide audience of the latter two.

A further issue with PCA is its inappropriateness with discrete data. MCA is one possible solution to this. The indices generated by PCA and MCA demonstrated high agreement, and had a very similar agreement with consumption expenditure. It therefore appears that, despite concerns over the violation of assumptions underlying PCA, using discrete data in a PCA-based wealth index is of limited cause for concern. Due to the advantages of PCA in terms of computational simplicity, we would not advocate the use of MCA in preference over PCA. Furthermore, continuous variables such as number of people per sleeping room or area of land owned cannot be included in MCA.

Despite the fact that PCA is unfamiliar to many readers of epidemiological research papers and that it could be accused of obscuring the process of constructing a wealth index, there seems to be little reason to adopt any of the alternatives explored in this analysis. Within the current study setting, the simpler methods resulted in indices with more clumping, and the inverse proportion method is unsuitable unless data collection has been preceded by substantial formative research. MCA is no simpler to implement or understand than PCA, cannot be used with a mixture of discrete and continuous variables, and results in an index with very high agreement with a PCA index. We would therefore recommend that having made the decision to construct a wealth index, PCA is a suitable tool for assigning weights to the indicators. Researchers are urged, however, to be clear about the concept of SEP they wish to measure, and to give careful consideration to the feasibility and appropriateness of alternative indicators such as consumption expenditure. The data used to construct a wealth index have a far stronger impact on the final wealth index than the method used to weight the items. Researchers planning data collection for a wealth index are therefore encouraged to carefully consider the data they collect rather than simply collecting data on the set of assets in DHS questionnaires. Formative research may help to identify assets that are strong predictors of SEP in a particular context, increasing the appropriateness of the wealth index as a measure of SEP. A further possibility for selecting assets for data collection is to identify assets which are highly correlated with consumption expenditure[40]. This approach requires full data on consumption expenditure and assets from a recent existing study in the same setting.

The difficulties of collecting income and consumption expenditure data for health research in low-income countries remain, and further alternatives to the wealth index approach are limited. Qualitative methods such as Participatory Wealth Ranking (PWR) have also been suggested as an alternative way of collecting SEP data, but such methods are probably only practical in small geographical areas[41-43]. This work has reviewed some of the issues with the wealth index approach to SEP measurement and has provided evidence that the data included in the index are more important than the method of index construction. We have also provided doubt that such an approach should be considered as a proxy for consumption expenditure, at least when using the standard set of assets collected by the DHS. This study, however, has been limited to a single dataset; further work to verify the generalisability of the findings in other contexts is recommended. In particular, results may differ in settings at varying stages of economic development. Furthermore, additional work on the consequences of using different sets of assets is recommended, as is an exploration of alternative methods to allow for price and other differences between urban and rural areas and between regions.

## Competing interests

The author(s) declare that they have no competing interests.

## Authors' contributions

LH designed the study, carried out data analysis and drafted the manuscript. JH contributed to study conception and design. SH supervised LH and contributed to study conception and design. All authors were involved in critical revision of the manuscript and read and approved the final manuscript.
